# Supplementary material for: The association between religiosity and resilience among young trans women
Source: PLoS One. 2023 Jul 31;18(7):e0263492. doi: 10.1371/journal.pone.0263492 (PMC10389706; doi:10.1371/journal.pone.0263492)
Supplement: Attachment — Submitted filename: Review of Wang et al._PLOS One_7-8-22.pdf [file pone.0263492.s001.pdf]

Review of "The association between religiosity and resilience among young trans women" Wang et al.

PLOS One

Comments to Authors

Overall this is well-structured and clearly written article. However, there are some issues that need to be addressed.

Major Revisions:

1. Since this is a cross-sectional study, cause and effect relationships cannot be determined. Therefore, religiosity may cause resilience **OR** resilience may cause religiosity **OR** both religiosity and resilience may be caused by a third or additional factors. The authors keep framing the association in this study as if religiosity causes resilience, in both the Introduction and Discussion sections. The authors should also consider and articulate other cause-and-effect relationships in both the Introduction and Discussion sections.
    a. For example, consider this scenario. This study was conducted in the SF Bay Area, a very wealthy area marked by liberal and progressive city and state policies that may be well-funded. Could it be that this economic/political environment promotes resilience in a variety of ways? And thus, already-resilient people are more likely to participate in social/civil society. And for Black young trans women (YTW), this greater participation may take the form of involvement in churches, hence, scoring high on religiosity.
2. In the Introduction section, the conceptual/theoretical construct for examining the possible association between religiosity and resilience is weak. Religiosity has a

psychological component but is also social and spiritual. Social support is barely mentioned and it would behoove the authors to further unpack how social support is fundamental to, and perhaps may even mediate, the association between religiosity and resilience.

a. If the authors choose to further unpack and discuss social support, then it would also be useful to discuss how social support can build social capital with respect to cultivating resilience. A landmark text on social capital is Putnam's (1995, 2000) work on bonding and bridging social capital. Hwahng et al., 2021, have discussed how social capital is related to resilience among trans women of color and have taken a more granular focus of "trust capital" within social capital, i.e. "thick trust" within bonding social capital and "thin trust" within bridging social capital. Given the intimate relationships (to a divine being and/or other members of a given faith community) inherent in religiosity, discussion of "trust capital" may be particularly salient to this article's premise. It would also be useful to include additional literature on social support and social capital within transgender communities such as Perez-Brumer et al., 2017; Erosheva et al., 2016; and Pinto et al., 2008. Finally, incorporating the "space of capabilities" construct (Sen, 1999) may also be useful since space of capabilities has implications for both resilience as well as religious practice.

3. The authors keep referring to "personal religiosity" in the Introduction and Discussion sections. Yet in the limitations the authors admit that the collected data was based on a question about the "importance of religion" in their lives and it is unknown if this

importance is due to participation in faith-based organizations and communities or "personal spirituality." And participation in faith-based organizations and communities could be characterized as social religiosity, cultural religiosity, and/or collective religiosity, which is distinct from "personal religiosity."

The authors thus need to clean up this language in the Introduction and Discussion sections and clearly state the actual research question that was asked in those sections, which is about the importance of religion, and not per se about "personal religiosity."

a. Given that the importance of religion for YTW may be due to past and/or current participation in faith-based organizations, it would be useful to conduct a more in-depth literature search on LGBTQ-inclusive and friendly faith-based organizations (including churches, mosques, temples, synagogues, etc.) and write at least one paragraph discussing these religious resources.
b. The authors could also conduct a more comprehensive lit review on LGBTQ people, resilience, and religion/spirituality. For example there has been a systematic review on religion, spirituality, mental health, and LGBTQ youth that was recently published (McCann et al., 2020) and a special issue on LGBTQ studies in *Psychology of Religion and Spirituality* (Yarhouse et al., 2021) that were not discussed.

4. It is unclear what the applications of this study may be, beyond just conducting more research. Are the authors interested in examining what factors may promote greater resilience for YTW? Or do the authors want barriers to be removed so that more YTW can be involved in faith-based organizations? Or are the authors interested in YTW

participating in greater civil society, more broadly defined? It would be useful for the reader to know what the overall intentions of the authors are in conducting this study, especially since causality cannot be established.

Minor Revisions:

1. On page 8, change heading from “Dependent Variable” to “Outcome of Interest” or another heading. This article is not merely a statistical exercise.
2. For Table 1, it would be useful to break down the categories of “Education” and “Monthly Income” by gender identity and race. For example, it might be of interest to note if certain racial or gender groups are more likely to have completed some college or have higher income compared to other racial or gender groups.
3. In Table 1, more space has to be included to separate the “Age” row from the rest of the table since the columns represent different measures for the “Age” row compared to the rest of the table.
4. In Tables 2 and 3, list adjusted variables at the bottom of these tables.
