# Supplementary material for: The association between religiosity and resilience among young trans women
Source: PLoS One. 2023 Jul 31;18(7):e0263492. doi: 10.1371/journal.pone.0263492 (PMC10389706; doi:10.1371/journal.pone.0263492)
Supplement: Attachment — Submitted filename: Review of Wang et al. REVISED_PLOS One_3-22-23.pdf [file pone.0263492.s003.pdf]

Review of "The association between religiosity and resilience among young trans women" Wang et al. REVISED

PLOS One

Comments to Authors

This revised version is a great improvement from the original version and the authors are commended for incorporating the feedback from the reviewers. A few minor revisions are suggested.

Minor Revisions:

1. There seems to be inconsistency throughout the article of either using "Black/African American" or "Black" to refer to the same racial identity, especially in the Discussion section. Please use only one of these racial identity labels consistently throughout the article.
2. In discussing the Results of Table 3 on page 16, it should also be mentioned that those who identified as "Other" reported less resilience compared to those who identified as 'Female," since this is a significant finding.
3. On page 20, lines 323-327, it is odd to briefly discuss that higher income was associated with increased resilience, since this association is not central to your research question. And it is unclear why the higher income association is discussed yet Hispanic/Latinx identity as a resilience association is not discussed in the Discussion section. Please either discuss both variables or neither variable.
4. On page 20, line 338, please write "an LGBTQ-affirming" instead of "a LGBTQ-affirming"
5. Given what is now written in the Introduction section on social capital fostering resilience, it seems that "social capital" could also be considered as a possible mediator.

Thus, “social capital” could be inserted on page 21, lines 350-351, before social support so that it now reads “(e.g., increased social capital, social support…”

6. “Hope” and “acceptance” on page 21, line 351 are vernacular terms that are not very much recognized scientifically. Can these terms be replaced with more scientifically recognized terms such as “well-being” and/or “positive outlook”?
