# Supplementary material for: The association between religiosity and resilience among young trans women
Source: PLoS One. 2023 Jul 31;18(7):e0263492. doi: 10.1371/journal.pone.0263492 (PMC10389706; doi:10.1371/journal.pone.0263492)
Supplement: Attachment — Submitted filename: Reviewer Responses_Shine_Religiosity_11_1_2022.docx [file pone.0263492.s002.docx]

Review of “The association between religiosity and resilience among young trans women”
Wang et al.
PLOS One

Dear Dr. DeBaets,

Thank you for the opportunity to revise and resubmit our research article entitled, “The association between religiosity and resilience among young trans women”. We have responded to all reviewer comments. In this draft, we followed the reviewers’ guidance to establish the theoretical basis for examining the potential association between religiosity and resilience, clarify language used to describe the “importance of religion” to participants, include additional information regarding existing trans-affirming faith-based institutions, describe additional literature on religion and resilience among trans youth, and emphasize the application of our study findings to improving resilience among young trans women. Also, we note additional limitations related to the cross-sectional design and lack of collection of information regarding religious affiliation of participants. We believe that these changes significantly strengthen the manuscript and thank the reviewers for their attention to our work.

Regarding data availability, the authors of this manuscript and the University of California, San Francisco IRB have ethical concerns related to sharing the study data publicly, given the small size of the transgender community in San Francisco. Sharing the primary data publicly may expose transgender women within the community and may place these participants at increased risk for violence and discrimination. Although de-identification of data typically renders participants unidentifiable, the small population of transgender women living in San Francisco means that even de-identified data poses a substantial risk to revealing participants’ identities. Therefore, it is our view and the view of our institution’s IRB that releasing the de-identified data publicly will place this already vulnerable population of young trans women at greater risk. Data requests can be made to the University of California, San Francisco IRB by phone: (415)476-1814 or email: IRB@ucsf.edu.

Sincerely,

The authorship team

Major Revisions:

**Reviewer 1
Comment 1:** *Since this is a cross-sectional study, cause and effect relationships cannot be
determined. Therefore, religiosity may cause resilience OR resilience may cause
religiosity OR both religiosity and resilience may be caused by a third or additional
factors. The authors keep framing the association in this study as if religiosity causes
resilience, in both the Introduction and Discussion sections. The authors should also
consider and articulate other cause-and-effect relationships in both the Introduction
and Discussion sections.*

*For example, consider this scenario. This study was conducted in the SF Bay
Area, a very wealthy area marked by liberal and progressive city and state
policies that may be well-funded. Could it be that this economic/political
environment promotes resilience in a variety of ways? And thus, already-resilient
people are more likely to participate in social/civil society. And for Black young
trans women (YTW), this greater participation may take the form of involvement
in churches, hence, scoring high on religiosity.*

**Response 1:**

Thank you for this comment. We agree that cause and effect cannot be established with cross-sectional data. As suggested, we have included additional text in the Introduction and Discussion sections to elaborate on the potential impact that resilience can have on religiosity on pages 5 and 22 (additions are highlighted below).

Page 5: “Second, this study is cross-sectional, limiting our ability to determine the temporal nature of the relationship between religiosity and resilience; namely, that being more religious causes increased resilience. It is also possible that individuals with greater resilience are more likely to participate in civil society, including in religious organizations, thus leading to increased religiosity.”

Page: 22 “In understanding the relationship between religiosity and resilience, it is also important to note that the relationship between religiosity and resilience is not necessarily unidirectional. A reciprocal relationship can exist, in which resilience enables greater civic engagement, including participation in religious practices and organizations.”

**Comment 2:** *In the Introduction section, the conceptual/theoretical construct for examining the
possible association between religiosity and resilience is weak. Religiosity has a
psychological component but is also social and spiritual. Social support is barely
mentioned and it would behoove the authors to further unpack how social support is
fundamental to, and perhaps may even mediate, the association between religiosity and
resilience.*

*If the authors choose to further unpack and discuss social support, then it would
also be useful to discuss how social support can build social capital with respect
to cultivating resilience. A landmark text on social capital is Putnam’s (1995,
2000) work on bonding and bridging social capital. Hwahng et al., 2021, have
discussed how social capital is related to resilience among trans women of color
and have taken a more granular focus of “trust capital” within social capital, i.e.
“thick trust” within bonding social capital and “thin trust” within bridging social
capital. Given the intimate relationships (to a divine being and/or other
members of a given faith community) inherent in religiosity, discussion of “trust
capital” may be particularly salient to this article’s premise. It would also be
useful to include additional literature on social support and social capital within
transgender communities such as Perez-Brumer et al., 2017; Erosheva et al.,
2016; and Pinto et al., 2008. Finally, incorporating the “space of capabilities”
construct (Sen, 1999) may also be useful since space of capabilities has
implications for both resilience as well as religious practice.*

**Response 2:**

Thank you for these recommendations. In the introduction section on pages 5-7, with additional review of the literature, we have included discussion on the pathways through which religiosity can influence resilience. We discuss the individual coping resources that some obtain through religiosity. We further discuss the expansion of social networks through participation in religious communities and highlight the potential impacts on social trust and social capital. Finally, we incorporate Sen’s “capability approach” to illustrate the impact that resilience and religious practice can have on individual wellbeing. To support these points, we have incorporated the reviewer’s suggested sources.

Pages 5-7: “Religiosity has been demonstrated to promote resilience through multiple pathways. Religiosity provides some people with coping resources such as benevolent religious reappraisal (i.e., finding positive meaning from an adverse event), coping self-efficacy (i.e., confidence in one’s ability to cope with adverse events), collaborative problem solving with a higher being, and seeking spiritual support from a higher being. (22,23)

Additionally, religiosity provides some with access to religious social networks that bolster resilience. Participation in religious organizations allows individuals to develop relationships with others who share similar spiritual and religious beliefs.(24) These social connections contribute to social capital, a group-level construct that includes networks, norms, and trust, which promotes coordinated action, and subsequently, collective access to social and material resources.(25)

Social trust is a cognitive domain of social capital which is strengthened by social interactions and allows groups of people to work together for a common goal. (26) Two types of social trust are theorized to exist. Thick *trust* refers to trust that is built on close relationships between individuals with shared social identity (e.g., family and friends) and thin trust refers to trust that is built on looser relationships with those of different social identities.(26) Thin trust is often developed through participation in formal institutions (e.g., community organizations and work).(26)

Research with trans women of color has revealed that thick trust builds social capital within groups by allowing members to receive emotional support from each other, express themselves authentically to each other, and receive tangible support (e.g., social, physical, and financial) from one another.(27) With a strong foundation of thick trust, some trans women feel empowered to accept and embrace differences of individuals within and outside of their groups, allowing them to build greater thin trust.(27) Thin trust enables some trans women to access resources previously unfamiliar to them and to work with organizations and other groups to expand access to medical, legal, and employment resources through advocacy and political action.(27)

Participation in religious communities and organizations provides individuals with access to diverse social networks that are bound together by a common spiritual identity, thus potentiating the development of thin and thick trust.(28) Ultimately, social trust fostered through participation in religious communities can facilitate development of social capital and greater resilience through increased access to social and physical resources.

Social capital has been demonstrated to bolster the resilience of trans women in a variety of settings. Trans women living with HIV have leveraged social capital within their communities to collaboratively overcome healthcare barriers, improve access to community health resources, and shift public stigma and discrimination.(29) Black and Latina transgender women have reported utilizing social capital to acquire emotional support, access gender affirming medical care, and organize civic and political action.(30) These resources, made available by social capital, help trans women overcome challenges generated by societal stigma and discrimination.

Resilience and participation in religious activities can independently contribute to an individual’s overall sense of wellbeing. This relationship can be understood through the “capability approach”, which details that wellbeing is dependent on an individual’s perceived ability to perform functions and activities that they value.(31) Inherent to the performance of most activities is some level of risk. Therefore, an individual’s resilience can help them to expand their capabilities by allowing them to overcome fear and bounce back from failure in pursuit of performing desired life functions.(32)

Under the framework of the “capability approach”, participation in religious and spiritual activities can represent an important function among those who value religion. Therefore, freedom to participate in religious communities and organizations as an individual desires can promote wellbeing.(33) Conversely, barriers to spiritual and religious practice, which many transgender people face, may be detrimental to wellbeing if religious practice is central to their cultural identity or value system.”

**Comment 3:** *The authors keep referring to “personal religiosity” in the Introduction and Discussion sections. Yet in the limitations the authors admit that the collected data was based on a question about the “importance of religion” in their lives and it is unknown if this importance is due to participation in faith-based organizations and communities or “personal spirituality.” And participation in faith-based organizations and communities could be characterized as social religiosity, cultural religiosity, and/or collective religiosity, which is distinct from “personal religiosity.” The authors thus need to clean up this language in the Introduction and Discussion sections and clearly state the actual research question that was asked in those sections, which is about the importance of religion, and not per se about “personal religiosity.”*

**Response 3:**

Thank you for this comment. We have modified text in the Introduction and Discussion sections on pages 8 and 19 to prevent conflation of “personal religiosity” with “religiosity” and the “importance of religion”. We consistently use “importance of religion” when referring to our data.

Page 8: “The primary goal of this study is to examine the association between the importance of religion and resilience among young trans women to determine if greater importance of religion could be a resilience promoting factor. The secondary goal is to identify demographic factors that are associated with importance of religion among young trans women.”

Page 19: “Our finding is consistent with qualitative research reporting that despite stigma from religious institutions, some transgender individuals identify religiosity to be a vital source of resilience.(45,46) Specifically, transgender participants from prior research have reported that their religiosity helps them to cope with adversity, foster hope, accept their personal identity, find their chosen family, and heal past trauma.(39–42,46)”

**Comment 4:** *Given that the importance of religion for YTW may be due to past and/or current
participation in faith-based organizations, it would be useful to conduct a more
in-depth literature search on LGBTQ-inclusive and friendly faith-based
organizations (including churches, mosques, temples, synagogues, etc.) and
write at least one paragraph discussing these religious resources.*

**Response 4:**

Thank you for this recommendation. On pages 21-22, we have included an in-depth review of existing LGBTQ inclusive religious organizations that support transgender individuals.

Pages 21-22: **“**Given the positive association between religiosity and resilience, efforts to support the spiritual needs of trans women may help to foster resilience. The importance of religion in the lives of some young trans women may stem from past or current participation in faith-based organizations. Within the United States, various LGBTQ-affirming places of worship exist. Mainstream religious denominations including the Unitarian Universalist Association, the United Church of Christ, the Episcopal church, Reform and Conservative Judaism, the Evangelical Lutheran Church in America, and the Presbyterian Church (USA) have officially adopted LGBTQ-inclusive policies.(47) Glide Memorial Church, in San Francisco, California has a long history of LGBTQ inclusion and advocacy and has hosted a Gender Expression & Identity summit, with the goal of fostering solidarity with the trans community and to hold space for trans individuals to express their spirituality.(48) Masjid al-Rabia is a LGBTQ-affirming Islamic community center in Chicago, Illinois that facilitates support groups for trans and gender nonconforming Muslims.(49) The Brooklyn Zen Center in Brooklyn, New York creates space for trans Buddhists, by hosting events that highlight the voices of trans Buddhist leaders.(50) While not comprehensive, these gender affirming faith-based organizations represent some of the religious communities that transgender people may participate in. Effort should be made to expand and support faith-based organizations like these, which serve as promoters of social capital and resilience for trans individuals. Additionally, transphobic doctrine and attitudes within all religious organizations must be reformed so that trans women can have greater access to this potential resilience promoting resource.”

**Comment 5:** *The authors could also conduct a more comprehensive lit review on LGBTQ
people, resilience, and religion/spirituality. For example there has been a
systematic review on religion, spirituality, mental health, and LGBTQ youth that
was recently published (McCann et al., 2020) and a special issue on LGBTQ
studies in* Psychology of Religion and Spirituality *(Yarhouse et al., 2021) that
were not discussed.*

**Response 5:**

Thank you for your referral to these sources. We carefully read the reviews and their primary source publications. We found none of the papers included in the McCann et al., 2022 review included transgender participants. Only one of the papers included in the Yarhouse et al., 2021 review includes transgender participants. These findings confirm the gap in the literature and the need for research specifically for transgender persons. While there are sometimes similarities between the experiences of sexual and gender minority youth, we understand that the experiences of transgender youth are often dissimilar to those of lesbian, gay, and bisexual youth. For example, even within LGB-affirming faith-based organizations, some transgender people have reported exclusion and discrimination due to their gender identity. As mentioned above, one of the papers (Gandy et al. 2021) in the Yarhouse et al. 2021 review includes interviews with trans youth. We have included discussion of the findings of this paper in the introduction section on page 8.

Page 8: “Some transgender people find their chosen family in supportive religious communities after facing rejection from their family of origin.(39) Furthermore, belief in a divine creator helps some transgender people to embrace and accept their identity.(40) In these supportive religious environments, transgender individuals report experiencing healing from past rejection and loss, inclusion, belonging, and joy.(39)”

**Comment 6:** *It is unclear what the applications of this study may be, beyond just conducting more research. Are the authors interested in examining what factors may promote greater resilience for YTW? Or do the authors want barriers to be removed so that more YTW can be involved in faith-based organizations? Or are the authors interested in YTW participating in greater civil society, more broadly defined? It would be useful for the reader to know what the overall intentions of the authors are in conducting this study, especially since causality cannot be established.*

**Response 6:**

Thank you for encouraging us to more boldly discuss the applications of our research. Our study highlights religiosity as a resilience resource for young trans youth. Based on this finding, we believe that support for existing trans-affirming faith-based organizations and the development of new affirming faith-based organizations that cater to the needs of trans women can promote resilience. Additionally, our findings support efforts to remove barriers to participation in existing faith-based organizations via reforming transphobic doctrine and attitudes. Application of study findings has been added to the Discussion section on page 22.

Page 22: “Effort should be made to expand and support faith-based organizations like these, which serve as promoters of social capital and resilience for trans individuals. Additionally, transphobic doctrine and attitudes within all religious organizations must be reformed so that trans women can have greater access to this potential resilience promoting resource.”

Minor Revisions: **Comment 1:** On page 8, change heading from “Dependent Variable” to “Outcome of Interest” or another heading. This article is not merely a statistical exercise.

**Response 1**: The heading has been changed to “Outcome of Interest” on page 11.

**Comment 2:** For Table 1, it would be useful to break down the categories of “Education” and
“Monthly Income” by gender identity and race. For example, it might be of interest to
note if certain racial or gender groups are more likely to have completed some college
or have higher income compared to other racial or gender groups.

**Response 2:** Thank you for this suggestion. We assessed the differences in demographic characteristics by gender identity and race/ethnicity and found that college education was higher among White compared to racial/ethnicity minority trans youth. We have incorporated this finding into the text. No other differences were found.

**Comment 3:**  In Table 1, more space has to be included to separate the “Age” row from the rest of the table since the columns represent different measures for the “Age” row compared to
the rest of the table.

**Response 3:** More space has been added after Age in Table 1 on page 13.

**Comment 4:** In Tables 2 and 3, list adjusted variables at the bottom of these tables.

**Response 4:** We have included the variables adjusted for in the footnotes of Tables 2 and 3 on pages 16 and 18.

**Reviewer 2**

**Comment 1:** *Does religious affiliation play a role? Some affiliations are more welcoming than others. Does your database have a variable for religious affiliation that you can also include in the model? This would allow you to see if your findings on the impact of personal religiosity vary by religious affiliation. For instance, you can interact the religious affiliation variable with the religious importance variable and use some effect plots to study this. If your database does not allow for this, then this marks another limitation of your analysis.*

**Response 1:** Thank you for this important question. Our survey did not collect information on the religious affiliation of participants. We recognize that religious affiliation may impact the way that religiosity influences resilience. Future studies should collect information on participant religious affiliation to better understand how it influences the relationship between religiosity and resilience. We have included this as a limitation of the study on page 23.

Page 23: “Fourth, information on religious affiliation with respect to religion, denomination, and degree of trans-affirmation was not collected from participants. The degree of trans-affirmation within a religious organization’s policies and culture may influence the effects that religiosity has on resilience of transgender members. Therefore, lack of consideration of religious affiliation of participants represents a limitation of this study.”

**Comment 2:** *Selection effects. The study assumes an exogenous relationship between religiosity and anti-gay attitudes. A religious institution’s stance on transgenderism (that is gender-affirming or not) could influence where transwomen worship and the importance they place on religion in their lives. The observed relationship could be due to selection effects, which marks a significant limitation of the findings that should be noted in the study.*

**Response 2:** Thank you for this observation. We recognize that the trans-affirmativeness of faith communities that trans women are a part of may be a confounder in the relationship between religiosity and resilience. We have included discussion of this in the limitations section on page 23.

Page 23: “Additionally, the degree of trans-affirmation of faith-based organizations that trans women participated in may independently influence their religiosity and resilience. Therefore, the observed association between religiosity and resilience within this study could theoretically be attributed to the confounding effects of the trans-affirmativeness of faith communities that participants were a part of. Future studies may examine the relationships between religiosity and resilience while controlling for trans-affirmativeness of faith-based communities that participants are members of.”
