# Supplementary material for: The association between religiosity and resilience among young trans women
Source: PLoS One. 2023 Jul 31;18(7):e0263492. doi: 10.1371/journal.pone.0263492 (PMC10389706; doi:10.1371/journal.pone.0263492)
Supplement: Attachment — Submitted filename: Shine Response PLOS_6_20_23.docx [file pone.0263492.s004.docx]

Dear Dr. Zhou,

Thank you for the opportunity to revise our manuscript. We appreciate the reviewers’ thorough comments. In this updated revision, we have modified the terminology used throughout the manuscript to improve consistency and accuracy. Additionally, we included reporting of all significant associations in the results section. We also removed discussion of topics that were not central to our research aims from the discussion section. We believe that these modifications significantly improve the clarity of our paper. Below is a point-by-point explanation of changes made to the revised manuscript.

Sincerely,

The authorship team

**Minor Revisions:**

1. There seems to be inconsistency throughout the article of either using “Black/African

American” or “Black” to refer to the same racial identity, especially in the Discussion

section. Please use only one of these racial identity labels consistently throughout the

article.

Thank you for this recommendation. To maintain consistency, we have decided to utilize “Black/African American” as this is the current official term used by the San Francisco Department of Public Health. This has been modified throughout the manuscript.

2. In discussing the Results of Table 3 on page 16, it should also be mentioned that those

who identified as “Other” reported less resilience compared to those who identified as

‘Female,” since this is a significant finding.

Thank you for this suggestion. We have included mention of “other” gender identity when discussing factors associated with resilience:

“Black/African American (aOR 4.25, 95% CI 1.56, 11.62, p=.005) and Hispanic/Latinx race/ethnicity (aOR 2.49, 95% 1.30, 4.77, p=.006), ‘other’ gender identity (aOR .29, 95% CI .09, .95, p=.04), and monthly income >$1,000 (aOR 3.87, 95% CI 2.00, 7.50, p=<.001) were also independently associated with increased resilience.”

3. On page 20, lines 323-327, it is odd to briefly discuss that higher income was associated

with increased resilience, since this association is not central to your research question.

And it is unclear why the higher income association is discussed yet Hispanic/Latinx

identity as a resilience association is not discussed in the Discussion section. Please

either discuss both variables or neither variable.

Thank you for this recommendation. We agree that the discussion of income is not central to our research aim. As such, we opted for your latter suggestion and have removed the paragraph mentioning income in the discussion section.

4. On page 20, line 338, please write “an LGBTQ-affirming” instead of “a LGBTQ-affirming”

This change has been made in the manuscript.

5. Given what is now written in the Introduction section on social capital fostering

resilience, it seems that “social capital” could also be considered as a possible mediator.

2

Thus, “social capital” could be inserted on page 21, lines 350-351, before social support

so that it now reads “(e.g., increased social capital, social support…”

Thank you for this recommendation. We have included social capital as a potential mediator: “Future studies are needed to identify factors that mediate the relationship between religiosity and resilience among transgender youth (e.g., increased social capital, social support, positive outlook, well-being)”

6. “Hope” and “acceptance” on page 21, line 351 are vernacular terms that are not very

much recognized scientifically. Can these terms be replaced with more scientifically

recognized terms such as “well-being” and/or “positive outlook”?

Thank you for this suggestion. We have modified the manuscript as follows: “Future studies are needed to identify factors that mediate the relationship between religiosity and resilience among transgender youth (e.g., increased social capital, social support, positive outlook, well-being)”
